# Cancer, platelet distribution width, and total protein levels as predictors of rebleeding in upper gastrointestinal bleeding

**DOI:** 10.7717/peerj.14061

**Published:** 2022-09-15

**Authors:** Ali Cankut Tatlıparmak, Özlem Dikme, Özgür Dikme, Hakan Topaçoğlu

**Affiliations:** 1 Department of Emergency Medicine, Kolan International Hospital, Istanbul, Turkey; 2 Department of Emergency Medicine, Istanbul Training and Research Hospital, Istanbul, Turkey; 3 Department of Emergency Medicine, Düzce University, Faculty of Medicine, Düzce, Turkey

**Keywords:** Upper gastrointestinal bleeding, Rebleeding, Predictors of rebleeding

## Abstract

**Background:**

Rebleeding is associated with poor outcomes in upper gastrointestinal bleeding (UGIB). Identifying predictors of rebleeding can assist in risk assessment. The aim of the study is to investigate the factors affecting rebleeding in patients with UGIB admitted to the emergency department.

**Methods:**

This retrospective, observational, cross-sectional study was conducted on patients with UGIB presented to the emergency department. Patients who did not arrest in the first 24 h, who were not diagnosed with GI malignancy, and who were clinically diagnosed with UGIB were included in the study. Patient demographic characteristics, hemodynamic parameters, patient parameters, and bleeding that may affect rebleeding were evaluated. The primary endpoint was rebleeding within 7 days.

**Results:**

The study included 371 patients. A total of 55 patients (14.8%) had rebleeding within 7 days, and 62 patients (16.7%) presented without bleeding manifestations. Rebleeding rates were higher in those who presented with bloody or coffee-ground vomitus, had a diagnosis of cancer, had blood in their nasogastric tube, and had peptic ulcers due to endoscopy. Mean cell hemoglobin concentration, lymphocyte, albumin, and total protein values of patients with rebleeding were low; red blood cell distribution width, neutrophil count, platelet distribution width (PDW), and neutrophil lymphocyte ratio were high. In-hospital mortality and 30-day mortality values of patients with rebleeding were significantly increased. In the multivariate analysis, cancer, PDW, and total protein levels were statistically significant.

**Conclusion:**

The presence of cancer, low total protein level, and high PDW are effective parameters in predicting 7-day rebleeding in patients with UGIB admitted to the emergency department.

## Introduction

Upper gastrointestinal bleeding (UGIB) is still a commonly encountered emergency, despite a reduction in incidence in the past 30 years. Through easy access to endoscopy, the usage rate of proton pump inhibitors and the eradication of *Helicobacter pylori* have increased (Abougergi, Travis & Saltzman, 2015). Even though etiologic and prognostic factors may have changed with increased direct anticoagulant use, rebleeding still plays an important role in mortality (Oakland, 2019; Quentin et al., 2021).

Patients with UGIB generally proceed to emergency medicine departments for their first medical contact. For these patients, risk assessment is also a part of the diagnosis and treatment process and is highly advised by consensus guidelines (Barkun et al., 2019). Several risk assessment tools and scores have been produced; however, their practical application is limited (Stanley et al., 2017). By contrast, changes in UGIB etiology may have also changed the predictors of rebleeding (Yue et al., 2021).

Even though UGIB is accepted as a single condition, it is, in fact, an umbrella clinical condition that consists of several underlying diseases (Nable & Graham, 2016). Definitive diagnosis is made by endoscopic evaluation in most cases; however, not all patients undergo endoscopy during emergency visit (Karadaş et al., 2020). Unless a patient is hospitalized, gastroenterologists needs to make a decision before performing an endoscopy (Siau et al., 2019). Therefore, determining the risk scores, classifying patients into either variceal or non-variceal cases, and identifying risk factors according to the definitive diagnosis are not practical tools in emergency medicine settings. In our study, we investigated the factors affecting rebleeding in patients with UGIB who presented to emergency medicine departments.

## Materials and Methods

### Study design and population

This study was a retrospective, observational, and single-center study conducted at the Health Sciences University, Istanbul Training and Research Hospital among patients consecutively admitted to the emergency department over 2 years. Institutional Review Board approval was obtained before the study by Istanbul Training and Research Hospital Ethics Committee (no: 1100).

### Selection criteria

The inclusion criteria were (a) over 18 years of age and (b) a clinical diagnosis of UGIB. UGIB was identified clinically in patients presenting with complaints suggesting UGIB after confirmation of diagnosis by examination and/or endoscopy. Trauma patients, patients who arrested within the first 24 h, patients with active lower gastrointestinal bleeding, patients with gastrointestinal cancer, or patients with insufficient information on the hospital system were excluded.

### Data collection

Patient demographics, chief complaints for emergency admission, blood pressure and heart rate at presentation, underlying diseases, history of medication (*i.e*., non-steroidal anti-inflammatory drugs [NSAIDs], steroids, anticoagulants, and antiplatelets), rectal examination findings, nasogastric tube contents, first laboratory results, Forrest score, bleeding origin, blood transfusion status, rebleeding rate, in-hospital mortality data, and 30-day mortality data were collected through the hospital information system.

Patients presenting to the emergency department with direct manifestations of UGIB (*i.e*., hematemesis or melena), as well as patients with indirect signs of blood loss (*i.e*., syncope, shock, dizziness, anemia, tachycardia, or pallor), were included if they were diagnosed with UGIB clinically and/or using endoscopy.

Patient hemodynamic status was reported as stable or unstable according to the first recorded data. Patients with systolic blood pressure above 90 mmHg and pulse rate lower than 100 beats/min was defined as stable, and those with systolic blood pressure above 90 mmHg or pulse rate greater than 100 beats/min was defined as unstable.

All patients were managed according to the UGIB treatment protocol of the hospital. Every patient in the study underwent endoscopy within 24 h, and the findings were collected in terms of Forrest classification (active spurting bleeding (1a), active oozing bleeding (1b), vessel exposure (2a), adherent clot (2b), and clean base ulcer (3)) (Forrest, Finlayson & Shearman, 1974) and the etiology of the bleeding.

Rebleeding was defined as hematemesis, hematochezia, and/or melena with hemodynamic instability or decreased hemoglobin to at least 2 g/dl within 24 h after the initial bleeding episode within 7 days after successful endoscopic therapy.

In-hospital mortality was defined as death during the index hospital stay, and 30-day mortality was defined as all-cause mortality within 30 days after the first emergency admission.

The primary outcome of the study was a rebleeding rate within 7 days after the first successful endoscopic intervention.

### Statistical analyses

Statistical analysis was performed using the SPSS 27.0 program for Windows (SPSS, Chicago, IL, USA). The normal distribution of variables was determined using the Shapiro–Wilk test. For descriptive statistics, continuous variables were presented as median values (interquartile range (IQR)), and nominal and categorical variables were presented as percentages or counts. Univariate analyses were performed using Fisher’s exact test, χ^2^ test, and independent sample t-test. Logistic regression analysis was performed to verify the results of the univariate analysis according to rebleeding rate using odds ratio (OR) and 95% confidence interval (CI). A 5-fold cross-validation was performed to evaluate model performance. The area under receiver operating characterics (AUROC) was performed for internal validation analysis. An alpha value of <0.05 was considered statistically significant (adjusted for multivariate analysis).

## Results

### Characteristics of patients

**Figure 1:**
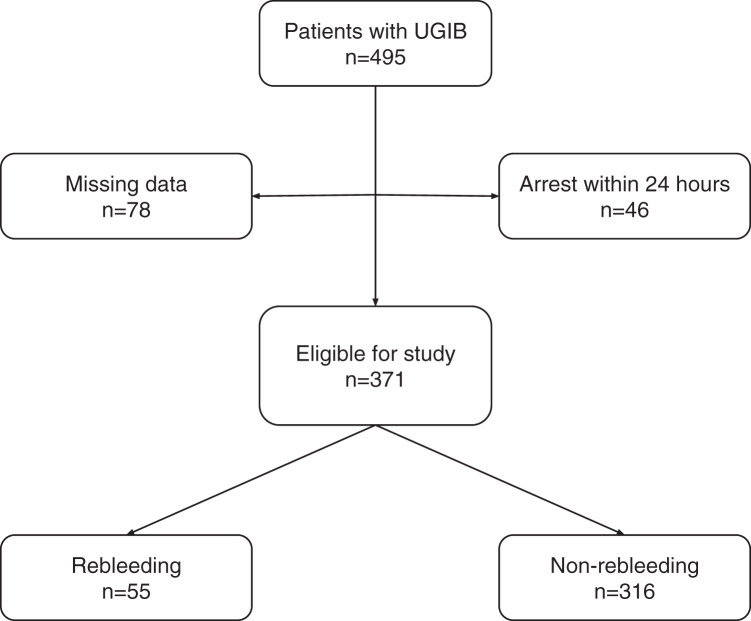
Patient flowchart.

As shown on Fig. 1; a total of 495 patients with UGIB over the age of 18 years were identified, and 124 were excluded due to early arrest (*n* = 46) or insufficient information (*n* = 78).

As shown in Table 1, a total of 371 patients (253 males, 118 females; median age 61 years, IQR 48–82) were analyzed. The patients were divided into two groups: the rebleeding and non-rebleeding patient groups. A total of 316 (85.2%) patients were in the non-rebleeding group, whereas 55 (14.8%) patients were included in the rebleeding group. The major chief complaints that led the patients to emergency were bloody and coffee-ground vomitus (*n* = 181, 48.8%) and dark tarry stool (*n* = 123, 33.2%). The rebleeding group showed high proportions of bloody and coffee-ground vomitus (*p* = 0.004). A total of 62 patients (16.7%) showed no bleeding. No patient with dizziness, palpitations, abdominal pain, or syncope encountered rebleeding. The most common co-morbidities were hypertension (*n* = 121, 32.6%) and diabetes mellitus (*n* = 61, 16.4%). Patients with cancer had a significantly high rate of rebleeding (*p* < 0.001). Among the groups, no significant differences in terms of NSAID, antiplatelet, or anticoagulant medication were found. In the rebleeding group (*n* = 14, 25.5%), a higher proportion of patients showed unstable hemodynamics at presentation than in the non-rebleeding group (*n* = 55, 17.4%); however, the difference was insignificant (*p* = 0.157). No significant difference in rectal examination findings was found among the groups; however, the rebleeding group had a higher proportion of patients with blood in the gastric tube than the non-rebleeding group (*p* = 0.019).

**Table 1:** Patient demographics and clinical data of patients with upper gastrointestinal bleeding.

	Non-rebleeding patients (*n* = 316, 85.18%)	Rebleeding patients (*n* = 55, 14.72%)	*p* value
Age (years)	61 (47–75)	63 (49–73)	0.807
Gender (male)	215 (68%)	38 (69.1%)	0.87
Co-morbidities			
Diabetes mellitus	54 (17.1%)	7 (12.72%)	0.424
Congestive heart failure	15 (4.75%)	6 (10.9%)	0.076
Liver disease	20 (6.33%)	4 (7.27%)	0.793
Hypertension	100 (31.6%)	21 (38.2%)	0.341
History of stroke	20 (6.3%)	4 (7.3%)	0.793
Chronic kidney disease	38 (12%)	7 (12.7%)	0.883
Cancer	21 (6.6%)	14 (25.5%)	**<0.001**
Medication			
NSAIDs	47 (14.9%)	4 (7.3%)	0.14
Antiplatelet	40 (12.7%)	5 (9.1%)	0.457
Anticoagulants	28 (8.9%)	3 (5.5%)	0.404
Chief complaint			
Dizziness	11 (3.5%)	0 (0%)	0.32
Palpitation	4 (1.27%)	0 (0%)	0.754
Fatigue	19 (6%)	5 (9.1%)	0.39
Bloody or coffee-ground vomitus	144 (45.6%)	37 (67.3%)	**0.004**
Rectal bleeding	4 (1.27%)	1 (1.81%)	0.744
Abdominal pain	11 (3.48%)	0 (0%)	0.325
Syncope	12 (3.80%)	0 (0%)	0.295
Dark tarry stool	111 (35.12%)	12 (21.82%)	0.056
Instable hemodynamics at presentation	55 (17.4%)	14 (25.5%)	0.157
Hematochezia	9 (2.8%)	3 (5.5%)	0.322
Melena	212 (67.1%)	37 (67.3%)	0.979
Blood in gastric tube	88 (27.8%)	24 (43.6%)	**0.019**
In-hospital mortality	1 (0.3%)	7 (12.7%)	**<0.001**
30-day mortality	5 (1.6%)	8 (14.5%)	**<0.001**

**Note:**

NSAIDs, non-steroidal anti-inflammatory drugs. Bold font indicates statistical significance.

In the laboratory findings at presentation (Table 2), mean cell hemoglobin concentration (*p* = 0.023), red cell distribution width (RDW; *p* = 0.003), neutrophil count (*p* = 0.013), lymphocyte count (*p* = 0.004), platelet distribution width (PDW; *p* = 0.003), albumin (*p* < 0.001), total protein (*p* = 0.006), and neutrophil-to-lymphocyte ratio (*p* < 0.001) were significantly different between groups. Both in-hospital mortality and 30-day mortality were significantly higher in the rebleeding group (*p* < 0.001).

**Table 2:** Lab results on presentation.

	Non-rebleeding patients (*n* = 316, 85.18%)	Rebleeding patients (*n* = 55, 14.72%)	*p* value
WBC (10^3^/µL)	9.53 (7.16–12.05)	10.44 (7.54–14.8)	0.085
HGB (g/dL)	9.6 (7.4–11.6)	8.65 (6.08–10.93)	0.076
PLT (10^3^/µL)	250 (187–323.5)	234 (180.3–321.3)	0.576
MCV (fL)	85.9 (82.2–89.6)	85.1 (79.3–91.1)	0.416
MCHC (g/dL)	32.6 (31.2–33.5)	32 (31.28–32.73)	**0.023**
RDW (%)	14.2 (13.3–15.9)	15.8 (13.85–18.25)	**0.003**
NEU (10^3^/µL)	6.33 (4.42–9.15)	8.02 (4.39–11.82)	**0.013**
LYM (10^3^/µL)	1.89 (1.25–2.62)	1.38 (0.86–2.27)	**0.004**
PDW (%)	15.8 (12.6–16.2)	16.1 (15.43–16.4)	**0.003**
Creatinine (mg/dL)	0.96 (0.77–1.31)	1 (0.8–1.32)	0.91
Urea (mg/dL)	63 (41.4–92.6)	64.65 (44.12–106.9)	0.36
Albumin (mg/dL)	3.44 (3.1–3.8)	3.1 (2.7–3.4)	**<0.001**
Total protein (mg/dL)	5.99 (5.45–6.4)	5.5 (5.01–6.15)	**0.006**
INR	1.1 (1–1.2)	1.1 (1–1.2)	0.339
NLR	3.34 (2.18–5.52)	5.5 (4.15–9.02)	**<0.001**

**Note:**

WBC, white blood cell count; HGB, hemoglobin; PLT, platelet; MCV, mean cell volume; MCHC, mean cell hemoglobin concentration; RDW, red blood cell distribution width; NEU, neutrophil; LYM, lymphocyte; PDW, platelet distribution width; INR, international normalized ratio; NLR, neutrophil-to-lymphocyte ratio. Bold font indicates statistical significance.

### Endoscopic findings

As shown on Table 3, in this study, the most common endoscopic finding was gastritis (*n* = 101, 27.2%). Interestingly, the most common endoscopic finding in the rebleeding group was peptic ulcers (*n* = 21, 38.1%), and the proportion was significantly higher than that in the non-rebleeding group (*p* = 0.012).

**Table 3:** Etiology of bleeding according to endoscopy.

	Non-rebleeding patients (*n* = 316, 85.18%)	Rebleeding patients (*n* = 55, 14.72%)	*p* value
Angiodysplasia	1 (0.3%)	1 (1.8%)	0.215
Duodenal ulcer	60 (19%)	12 (21.8%)	0.625
Gastritis	94 (29.7%)	7 (12.7%)	**0.012**
Mallory–Weiss	7 (2.2%)	0 (0%)	0.5
Peptic ulcer	70 (22.2%)	21 (38.1%)	**0.012**
Esophageal ulcer	3 (0.9%)	1 (1.8%)	0.572
Esophageal varices	29 (9.2%)	5 (9.1%)	0.984
Esophagitis	8 (2.5%)	0 (0%)	0.445
No findings	44 (13.9%)	8 (14.5%)	0.90

**Note:**

Bold font indicates statistical significance.

### Multivariate analysis

Given that significant differences were found between the groups in the above-mentioned parameters through univariate analysis, a multivariate stepwise forward logistic regression analysis was conducted (Table 4). The Hosmer-Lemeshow goodness of fit test showed a χ^2^ value of 6.94 and a *p* value of 0.543. The model explained 24,4% of variance in rebleeding (Nagelkerke R^2^). The performance of the model yielded an accuracy of 84,9% and area under curve (AUC) value of 0.754 (95% CI [0.683–0.826]). Analysis revealed that PDW (*p* = 0.006, OR = 1.31, 95% CI [1.08–1.59]), total protein (*p* = 0.045, OR = 0.51, 95% CI [0.27–0.99]), and cancer (*p* = 0.015, OR = 3.64, 95% CI [1.28–10.3]) were independent predictive variables for rebleeding in UGIB. A 5-fold cross validation was performed to analyze the model (Table 5).

**Table 4:** Multivariate analysis results of rebleeding.

	*p* value	OR	95% CI
PDW	0.006	1.31	[1.08–1.59]
Total protein	0.045	0.51	[0.27–0.99]
Cancer	0.015	3.64	[1.28–10.3]

**Note:**

PDW, platelet distribution width; OR, odds ratio; CI, confidence interval.

**Table 5:** Cross-validation of model.

	Accuracy (%)	AUC	*p* value
Complete data set	84.9	0.754 (0.683–0.826)	<0.001
Fold 1	79	0.807 (0.687–0.927)	<0.001
Fold 2	88.6	0.783 (0.627–0.939)	0.004
Fold 3	85.2	0.718 (0.536–0.899)	0.038
Fold 4	91.5	0.813 (0.593–0.999)	0.012
Fold 5	86.6	0.635 (0.526–0.764)	0.034

**Note:**

AUC, Area under the curve.

## Discussion

This retrospective observational study revealed that elevated PDW and total protein levels and the presence of cancer are independent risk factors in predicting rebleeding in patients admitted to the emergency department and diagnosed with UGIB.

In our study, the rate of rebleeding was 14.72%, which is consistent with the literature (Fukuda et al., 2020; Yue et al., 2021; Quentin et al., 2021). In most studies, the definition of rebleeding was consistent; however, the durations determined for this definition differed from each other. Even though the long periods determined regarding rebleeding in the studies conducted in the emergency department may contain unrealistic expectations, in the expression of rebleeding before discharge without specifying the duration, comorbid diseases that prolong the hospitalization period may be more associated with rebleeding than that observed in reality.

Although UGIB often manifests bleeding, it can sometimes be diagnosed by indirect signs of bleeding (The Initiatives de Recherche aux Urgences Group et al., 2017). In our study, 62 patients (16.7%) applied to the emergency department with indirect signs of bleeding and were diagnosed with UGIB with the aid of a physical examination and/or endoscopy. In our study, both the admission with a complaint of bloody or coffee-ground vomiting and the presence of blood in the nasogastric tube were statistically significantly high in terms of rebleeding. Given that the current UGIB guidelines do not recommend the routine use of nasogastric tubes, nasogastric tubes were administered to patients at the discretion of the primary physician (Barkun et al., 2019). Even though this can be considered a selection bias, bloody nasogastric tube content is associated with poor prognosis in the literature (Aljebreen, Fallone & Barkun, 2004; Palamidessi et al., 2010); and in our study, the application of nasogastric tube on patients with bloody or coffee-ground vomitus showed significantly high rebleeding occurrence.

Oncology patients are highly likely to have GI bleeding (Yarris & Warden, 2009; Maluf-Filho et al., 2013; Minhem et al., 2021). According to a study conducted with 640,000 patients with GI bleeding, cancer has been observed at a rate of 4.76% in patients with GI bleeding; by contrast, this rate was higher in our study (9.4%) (Minhem et al., 2021). The fact that our study was conducted in a major oncology center may play an important role in this finding. Among cancer patients, GI bleeding was most frequently detected in patients with gastric cancer. The most common cause of bleeding in patients with gastrointestinal tract tumors has been reported as tumor bleeding (Maluf-Filho et al., 2013). When patients with gastrointestinal system tumors were excluded, the etiologies of UGIB were actually similar to the population. In our study, the presence of cancer was identified as an independent risk factor for rebleeding. No other study investigating the relationship between rebleeding and cancer has been found in the literature; however, cancer patients have a predisposition to bleeding. We hypothesize that the factors that predispose patients to bleeding may also play a role in rebleeding.

Although peptic ulcer disease is the most common cause of UGIB in the literature, gastritis was observed more often than peptic ulcers in our study (Oakland, 2019). Even though previous studies have shown no association between gastric ulcers or gastritis and rebleeding (Yue et al., 2021), the rate of endoscopic diagnosis of peptic ulcers was found to be significantly high in patients with rebleeding, and the rate of diagnosis of gastritis was found to be significantly high in patients who showed no rebleeding.

Platelet indices are important for primary hemostasis. Platelet parameters can be summarized as plateletcrit, MPV, and PDW. Inarguably, approximately 80% of UGIB cases stop spontaneously by primary hemostasis (van Leerdam et al., 2003). However, platelets also play an active role in wound healing after primary hemostasis. Senel et al. (2019) found that PDW is the platelet parameter most associated with UGIB. No other publications investigating the relationship between PDW and rebleeding have been found in the literature. In our study, rebleeding was observed as an independent risk factor, according to the PDW multivariate analysis.

Total protein was found to be lower in UGIB than in lower GI bleeding, and this factor may be used to differentiate between upper and lower GI bleeding (Tomizawa et al., 2015). No other study about the relationship between total protein and rebleeding in UGIB has been found in the literature. In our study, decreased total protein was observed as a predictor of rebleeding in UGIB. However, its mechanism of action remains unclear.

The reason we used rebleeding as the primary endpoint in our study was that rebleeding is directly associated with mortality in previous studies (Quentin et al., 2021). In our study, rebleeding was associated with both in-hospital mortality and 30-day mortality.

### Limitations

This study has several limitations. It is a single center, observational, retrospective study. Due to the nature of the condition; it is impossible to determine the exact time bleeding has started and time elapsed between the start of bleeding and presentation to emergency department may vary among patients. The sample size is small (*n* = 371) and the data is imbalanced (14.8% in the rebleeding group) for a precise multivariate analysis. Without a separate validation set to replicate these findings; it is hard to conclude PDW, total protein and cancer as independent prognostic factors.

## Conclusions

In UGIB, rebleeding is closely associated with mortality. According to our study, PDW, total protein, and cancer are associated with rebleeding in UGIB; and could be considered in future prognostic model developments that might be required with changing etiology.

## Supplemental Information

10.7717/peerj.14061/supp-1 Supplemental Information 1 Dataset.

## Additional Information and Declarations

### Data Availability

The following information was supplied regarding data availability: The raw data are available in the Supplementary File.
